# Characterization, expression patterns of molt-inhibiting hormone gene of *Macrobrachium nipponense* and its roles in molting and growth

**DOI:** 10.1371/journal.pone.0198861

**Published:** 2018-06-11

**Authors:** Hui Qiao, Fengwei Jiang, Yiwei Xiong, Sufei Jiang, Hongtuo Fu, Fei Li, Wenyi Zhang, Shengming Sun, Shubo Jin, Yongsheng Gong, Yan Wu

**Affiliations:** 1 Key Laboratory of Freshwater Fisheries and Germplasm Resources Utilization, Ministry of Agriculture, Freshwater Fisheries Research Center, Chinese Academy of Fishery Sciences, Wuxi, China; 2 Wuxi Fisheries College, Nanjing Agricultural University, Wuxi, China; Shanghai Ocean University, CHINA

## Abstract

The oriental river prawn, *Macrobrachium nipponense*, is an important commercial aquaculture resource in China. In order to overwinter, *M*. *nipponense* displays decreased physiological activity and less consumption of energy. Sudden warming would trigger molting and cause an extensive death, resulting in huge economic losses. Therefore, it is of great practical significance to study the molting mechanism of oriental river prawns. Molt-inhibiting hormone gene (MIH) plays a major role in regulating molting in crustaceans. In this study, a full length MIH cDNA of *M*. *nipponense* (Mn-MIH) was cloned from the eyestalk. The total length of the Mn-MIH was 925 bp, encoding a protein of 119 amino acids. Tissue distribution analysis showed that Mn-MIH was highly expressed in the eyestalk, and that it had relatively low expression in gill, ovary, and abdominal ganglion. Mn-MIH was detected in all developmental stages, and changed regularly in line with the molting cycle of the embryo and larva. Mn-MIH varied in response to the molting cycle, suggesting that Mn-MIH negatively regulates ecdysteroidogenesis. Mn-MIH inhibition by RNAi resulted in a significant acceleration of molting cycles in both males and females, confirming the inhibitory role of MIH in molting. After long-term RNAi males, but not females had significant weight gain, confirming that Mn-MIH plays an important role in growth of *M*. *nipponense*. Our work contributes to a better understanding of the role of Mn-MIH in crustacean molting and growth.

## Introduction

The oriental river prawn *Macrobrachium nipponense* (Decapoda, Palaemonidae), which is maily distributed in East Asia, is an important commercial resource for aquaculture in China. It is especially popular in east China [1]. The latest economic data on these spices indicate an annual culture production of about 272,592 tons with a value near 20 billion RMB in 2016 [2]. Due to winter’s low temperature, every year prawns enter into a slow development process, characterized by decreased physiological activity and less consumption of energy. Sudden warming can trigger molting and cause extensive death. It increased risk of breeding and caused incalculable economic damage to fishermen, and also blocked breed selection and conservation. Therefore, it is of great practical significance to study the molting mechanism of oriental river prawns, which would help to make genetic improvements and improve production.

Molting plays a crucial role in development of crustaceans, in their growth, and in their reproduction. During their lifetime, crustaceans undergo precise physiological processes of molting, degrading the old exoskeleton, and synthesizing a new exoskeleton [3]. Molting in crustaceans is mainly controlled by ecdysteroids. It is assumed that, during the molting cycle of crustacean, MIH secreted from the X-organ/sinus gland complex inhibits the ecdysteroid synthesis in Y-organs and suppresses molting [4–7]. In crustaceans, MIH and members of the family of crustacean hyperglycemic hormone (CHH) neuropeptide, including crustacean hyperglycemic hormone (CHH) and gonad inhibiting hormone (GIH), have been widely investigated [8–9]. These hormones are involved in many biological processes, such as molting, reproduction, glucose metabolism, morphogenesis and osmoregulation [10–13]. Excision of the eyestalk and inhibition of MIH genes by RNA interference (RNAi) can therefore accelerate molting and gonad maturation [12, 14–17]. In *M*. *nipponense* studies on MIH are still scarce. Studies on growth regulation by MIH in crustaceans are unavailable.

In this study, we cloned a full length MIH gene from the eyestalk of the oriental river prawn *M*. *nipponense*, and analyzed its sequence features. In addition, the relative expression of Mn-MIH in various tissues and development stages of the embryo was assessed. Then we investigated the expression patterns of Mn-MIH during molting cycles. Short-term RNAi was used to demonstrate the function of Mn-MIH in molting progress. Furthermore, long-term RNAi was used to clarify the role of MIH in growth and development. Our work will contribute to a better understanding of regulatory mechanisms involved in crustacean molting and shed further light on genetic improvement in this commercially important prawn species.

## Materials and methods

### Ethics statement

All experiments involving *M*. *nipponense* in this study have been approved by Institutional Animal Care and Use Ethics Committee of the Freshwater Fisheries Research Center, Chinese Academy of Fishery Sciences (Wuxi, China).

### Animals

Healthy pawns in the experiments, approximately2.34±0.66 g in wet weight, were get from Freshwater Fisheries Research Center, Chinese Academy of Fishery Sciences, Wuxi, China and cultured in a recirculating water aquarium system and fed with paludina twice per day.

### RNA extraction and cDNA synthesis

Total RNA was isolated from mixed eyestalks of prawns using RNAiso Plus Reagent (TaKaRa, Japan) according to the manufacturer's protocols. The quality and concentration of the extracted RNA were checked by 1.2% agarose gel electrophoresis and a NanoDrop 1 ND2000 (NanoDrop Technologies Inc., Wilmington, DE, USA). First strand cDNA was synthesized using Reverse Transcriptase M-MLV Kit (TaKaRa, Japan). Finally, the cDNAs were stored at −80°C until further processing.

### Cloning and bioinformatics analysis of Mn-MIH

The primers, FZ and RZ (Table 1), to recognize the MIH sequence, were designed based on the amino acid sequences of the *M*. *rosenbergii* MIH gene (GenBank accession number: KC990939.1). PCR amplifications were performed according to reference [8]. The PCR products were purified using Gel Extraction kit (Sangon, China) and sequenced after insertion into PMD-18T vector. An amplicon of 554 bp was obtained and confirmed by sequencing as a result.

**Table 1:** Primers used in this study.

Primer name	Sequence(5′→3′)	Purpose
**Mn-MIH F1**	CCGGCATTCCTTTGTTTACCTG	Homology-based clone primer
**Mn-MIH R1**	ATATTCTGGCGTGTGGTCCTG	Homology-based clone primer
**Mn-MIH F2**	CCTGAGTGTCTGTCCAGTTTGA	Homology-based clone primer
**Mn-MIH R2**	TGGTCCTGGAGCTTATTTTAGAGG	Homology-based clone primer
**Mn-MIH-3GSP1**	TAGATGACGAATGCCCTGGTG	FWD1st primer for 3′ RACE
**Mn-MIH-3GSP2**	TGGACTTCTTGTGGTGCGTCTAC	FWD 2nd primer for 3′ RACE
**Mn-MIH-5GSP1**	AACAATCGTCGCATACCCTGA	RVS 1st primer for 5′ RACE
**Mn-MIH-5GSP2**	GGTTTACCATTCCTTGCAGGTG	RVS 2nd primer for 5′ RACE
**3′ RACE Outer**	TACCGTCGTTCCACTAGTGATTT	FWD 1st primer for 3′ RACE
**3′ RACE Inner**	CGCGGATCCTCCACTAGTGATTTCACTATAGG	FWD 2nd primer for 3′ RACE
**5′ RACE Outer**	CATGGCTACATGCTGACAGCCTA	RVS 1st primer for 5′ RACE
**5′ RACE Inner**	CGCGGATCCACAGCCTACTGATGATCAGTCGATG	RVS 2nd primer for 5′ RACE
**Mn-MIH-RTF**	AGCACCTGCAAGGAATGGTAA	FWD primer for Mn-MIH expression
**Mn-MIH-RTR**	AACAGTCCTTCCTGCATCTGG	RVS primer for Mn-MIH expression
**β-actinF**	TATGCACTTCCTCATGCCATC	FWD primer for β-actin expression
**β-actinR**	AGGAGGCGGCAGTGGTCAT	RVS primer for β-actin expression
**Mn-MIH -dsF**	TAATACGACTCACTATAGGGGTAAACCTGACAGCGCAAGG	For Mn-MIH dsRNA synthesis
**Mn-MIH -dsR**	TAATACGACTCACTATAGGGCCATCTGTTGAGCTGTTCCA	For Mn-MIH dsRNA synthesis
**GFP -dsF**	TAATACGACTCACTATAGGGACGAAGACCTTGCTTCTGAAG	For GFP dsRNA synthesis
**GFP -dsR**	TAATACGACTCACTATAGGGAAAGGGCAGATTGTGTGGAC	For GFP dsRNA synthesis

To carry out a rapid amplification of the cDNA ends (5’ and 3’ RACE), specific primers (Table 1) were designed based on the obtained partial cDNA sequence. The 3′-RACE and 5′-RACE were performed using 3′-full RACE Core Set Ver.2.0 Kit and 5′-full RACE Kit (TaKaRa, Japan) to get cDNA 3’ and 5’ ends according to the protocol and the PCR amplifications were performed according to the reference [8]. The PCR products were electrophoresed on a 1.5% agarose gel, and purified with the Gel Extraction kit (Sangon, China). The expected purified fragments were cloned into the PMD-18T vector, and positive clones were selected and sequenced by bABI3730 DNA Analyzer. Primers Mn-MIH F and Mn-MIH R were used to validate the open reading frame (ORF) using the following PCR program: denaturation at 94°C for 3 min, 30 amplification cycles of denaturation at 94°C for 30 s, annealing at 55°C for 30 s and elongation at 72°C for 2 min, and a final elongation at 72°Cfor10 min.

The sequences from RACEs were assembled by DNAMAN 6.0.40. The full length sequence was analyzed based on the nucleotide and protein databases using the BLASTX and BLASTN program (http://www.ncbi.nlm.nih.gov/BLAST).The signal peptides were predicted by SignalP3.0 (http://www.cbs.dtu.dk/services/SignalP/) using both nearest-neighbor and hidden Markov model (HMM) algorithms. The neighbor-joining method was used to construct the phylogenetic tree by Molecular Evolutionary Genetics Analysis (MEGA5) and bootstrapping replications were 5000. The nucleotide sequences of MIHs, GIHs and CHHs from other crustaceans were downloaded from the GenBank database (http://www.ncbi.nlm.nih.gov/).

### Real-time quantitative PCR analysis

The quantitative real-time PCR was used to detect the expression profile of Mn-MIH in different tissues, embryo developmental stages and molting cycles. It was also used in relative mRNA expression detection of RNAi experiment. The quantitative real-time PCR assay were performed on Bio-Rad iCycler iQ5 Real-Time PCR System (Bio-Rad,USA). Amplifications were performed with a 25 μL reaction volume containing 50ng cDNA template, 10μL SsoFastTM EvaGreen® Supermix (BIO-RAD, CA, USA), 0.5μL 5μM of Mn-MIH -qF and Mn-MIH -qR primers, and 13 μL of dd-water. The PCR temperature profile was performed according the previous study [8]. A Mn-β-Actin was used as a the internal control gene. All the primers used were listed in the Table 1. Each sample was analyzed in triplicate and at least three samples at the same stage were processed. The dissociation curve analysis of amplification products was performed at the end of each PCR reaction to ensure that only one PCR product was amplified and detected. The relative copy number of Mn-MIH mRNA was calculated using the 2^-⊿⊿CT^ comparative CT method [18].

### RNA interfering

dsRNA was synthesized using the MEGAscript T7 Kit (Ambion, Foster City, CA, USA). dsRNA of Mn-MIH was synthesized using Transcript AidTM T7 High Yield Transcription kit (Fermentas, Inc., USA). Green Fluorescent Protein gene (GFP) was used as a control. Primers used in RNAi were designed according to the cDNA sequences of Mn-MIH and GFP using SnapDragon-dsRNA Design (http://www.flyrnai.org/cgi-bin/RNAi_find_primers.pl). Templates for in vitro transcription were prepared by PCR using gene-specific primers Mn-MIH iF, Mn-MIH iR and GFP-iF, GFP-iR with the T7 polymerase promoter sequence at their 5′ ends (Table 1). dsRNA quality was assessed by 1.5% agarose gel electrophoresis, and the concentration of dsRNA was measured at 260 nm using a BioPhotometer (Eppendorf, Hamburg, Germany). The dsRNA was then kept at − 20°C until use.

The RNAi experiment was carried out in Nov. to Dec in 2016. All prawns were cultured at water temperature 25°C ± 1°C. For the short-term dsRNA injection experiment, 50 adult healthy prawns (weights of 1.50±0.35 g) were randomly assigned to two groups. The experimental group (n = 25) was injected with MIH-dsRNA. Each prawn was injected with MIH-dsRNA through the pericardial cavity membrane of the carapace at a dose of 4μg g^−1^.b.w (based on gram body weight). The control group (n = 25) was injected with GFP-dsRNA at volumes equivalent to those applied to the experimental group. Three prawns from each group were randomly collected at 2^nd^, 4^th^, 6^th^, 8^th^, 10^th^ and 12^th^ after injection. The Mn-MIH expression of the eyestalks was investigated to detect the interference efficiency by qPCR.

For long-term RNAi experiment, 60 males and females *M*. *nipponense* (body weight 0.66±0.16 g) at the same developmental stage were divided into four groups, including MIH-dsRNA injected males (n = 15), GFP-dsRNA injected males (n = 15), MIH-dsRNA injected females (n = 15), and GFP-dsRNA injected females (n = 15). Each prawn was injected with 4 μg g^−1^.b.w ds RNA or the same volume of vehicle every week. The total number of molting shells were counted every day during the 6 weeks, and average body weight data was documented at beginning and end of the experiment.

### Statistical analysis

All quantitative data were presented as mean ± SD. The differences between groups were analyzed using one-way ANOVA followed by Duncan's multiple range tests with the SPSS 20.0 software (IBM, New York, NY, USA). The significance level of data variance was set at 0.05. Chi-square and T test was used to compare expression levels and shells, body weights in RNAi.

## Results

### Isolation and characterization of Mn-MIH

**Fig 1:**
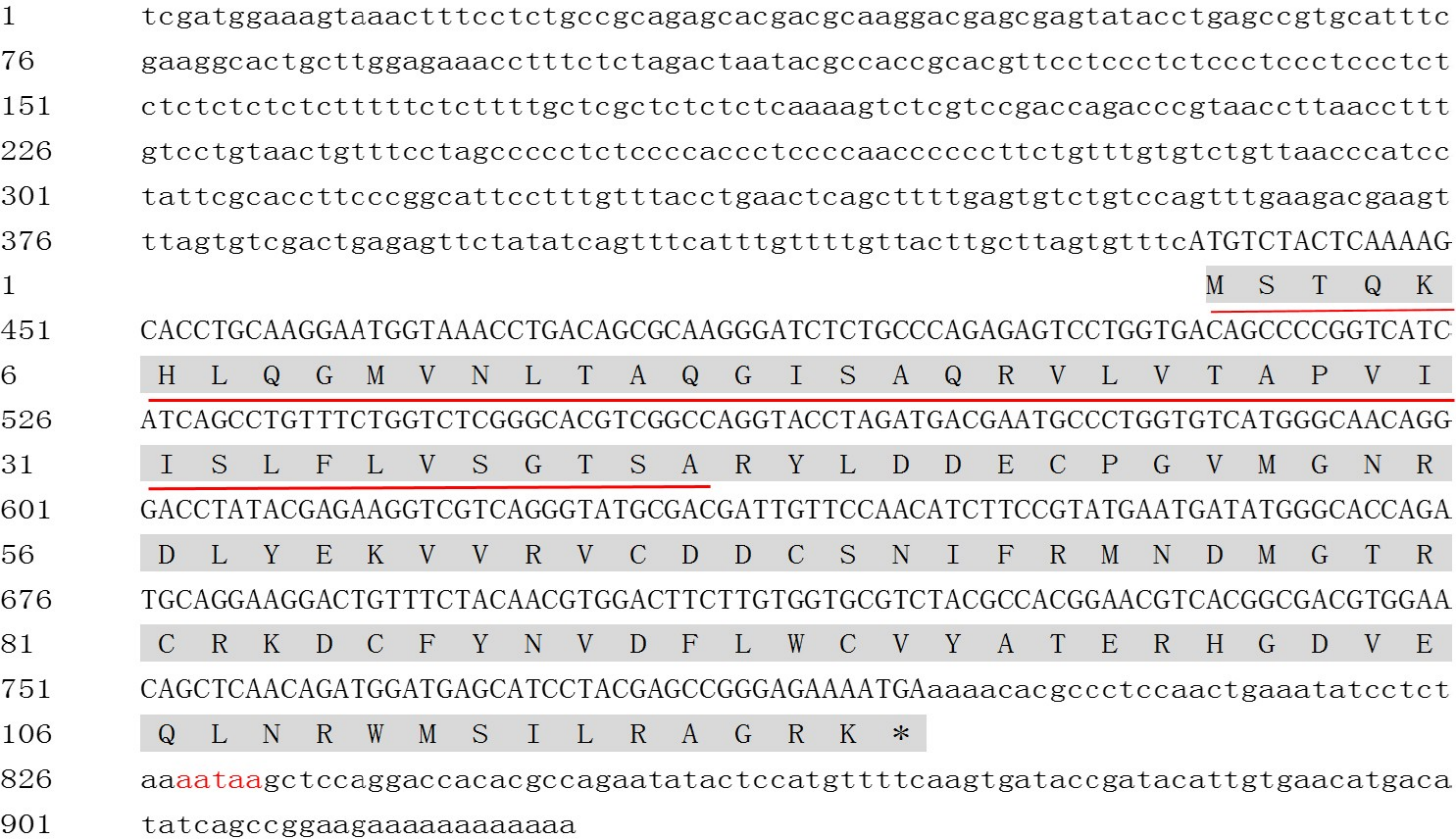
Nucleotide and deduced amino acid sequences of Mn-MIH. Notes: The numbers on the left of the sequences show the coordinate of nucleotides; the amino acids are presented as one-letter symbols and shown below their codons in each line and termination of amino acids is denoted by asterisks; signal peptide is single underlined; putative polyadenylation signal (AATAAA) is double underlined.

**Fig 2:**
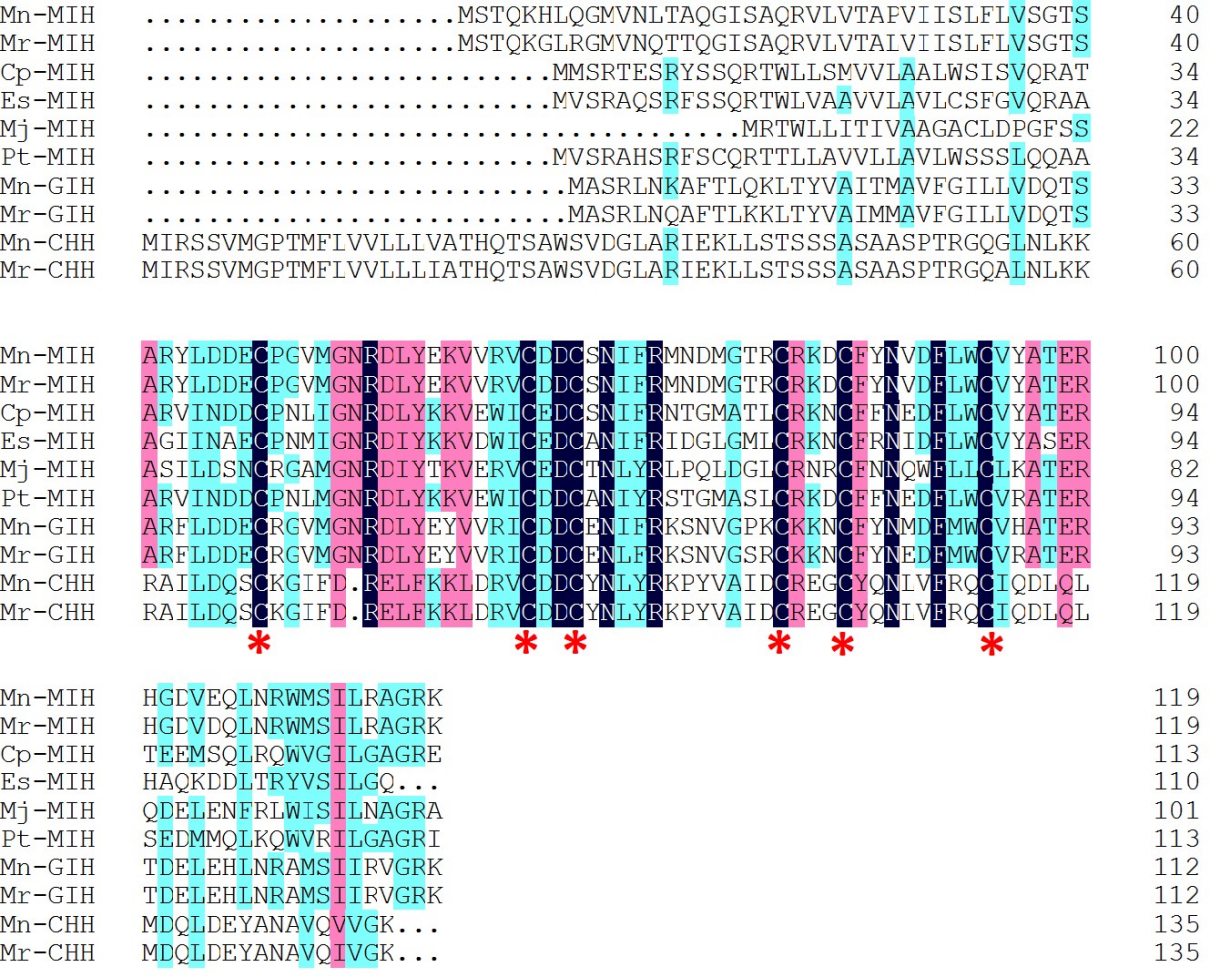
Alignment of deduced amino acid sequence of Mn- MIH with other CHH family neuropeptides. Notes: The deduced amino acid sequence of MIH from *M*. *nipponense* (Mn-MIH, in this study) is aligned with MIHs of MIHs of *Macrobrachium rosenbergii* (Mr-MIH; AAL37948.1), *Cancer pagurus* (Cp-MIH;CAC05346.1), *Eriocheir sinensis* (Es-MIH; AAQ81640.1), *Marsupenaeus japonicus* (Mj- MIH; BAE78494.1), *Portunus trituberculatus* (Pt-MIH;ABZ04547.1), and GIH of *M*. *nipponense* (Mn-GIH; AEJ54623.1), *M*. *rosenbergii* (Mr-GIH; AAL37949.1) CHHs of *M*. *nipponense* (Mn-CHH; AEJ54624.1), *M*. *rosenbergii* (Mr-CHH; AAL40915.1). Sequence identities are highlighted in black color, and pink depicts the conservative changes. Asterisks denoted conservation of cysteines.

Degenerated PCR generated an amplicon of 554 bp. The full length Mn-MIH cDNA was 925 bp (GenBank accession no. KF878973), containing a 435 bp 5′untranslated region (UTR), a 360 bp open reading frame and a 130 bp 3′UTR with a single typical polyadenylation signal (AATAAA) upstream from the poly (A) tail (Fig 1). The full length Mn-MIH encoded a putative protein of 119 amino acid residues with a predicted molecular weight of 13.60 kDa, and an isoelectric point of 8.26. Sequence analysis using Signal 3.0 Server predicted a signal peptide of 41 amino acids which would yield a mature peptide of 78 amino acids. The Mn-MIH protein contains six conserved cysteines (Cys7, Cys24, Cys27, Cys40, Cys44, Cys53) with three intrachain disulfide bonds (Fig 2). All of these, as well as a specific glycine of the CHH family II of peptides (Gly^46^), are conserved across the CHH family of peptides in crustaceans.

### Sequence comparison and phylogenic analysis

**Fig 3:**
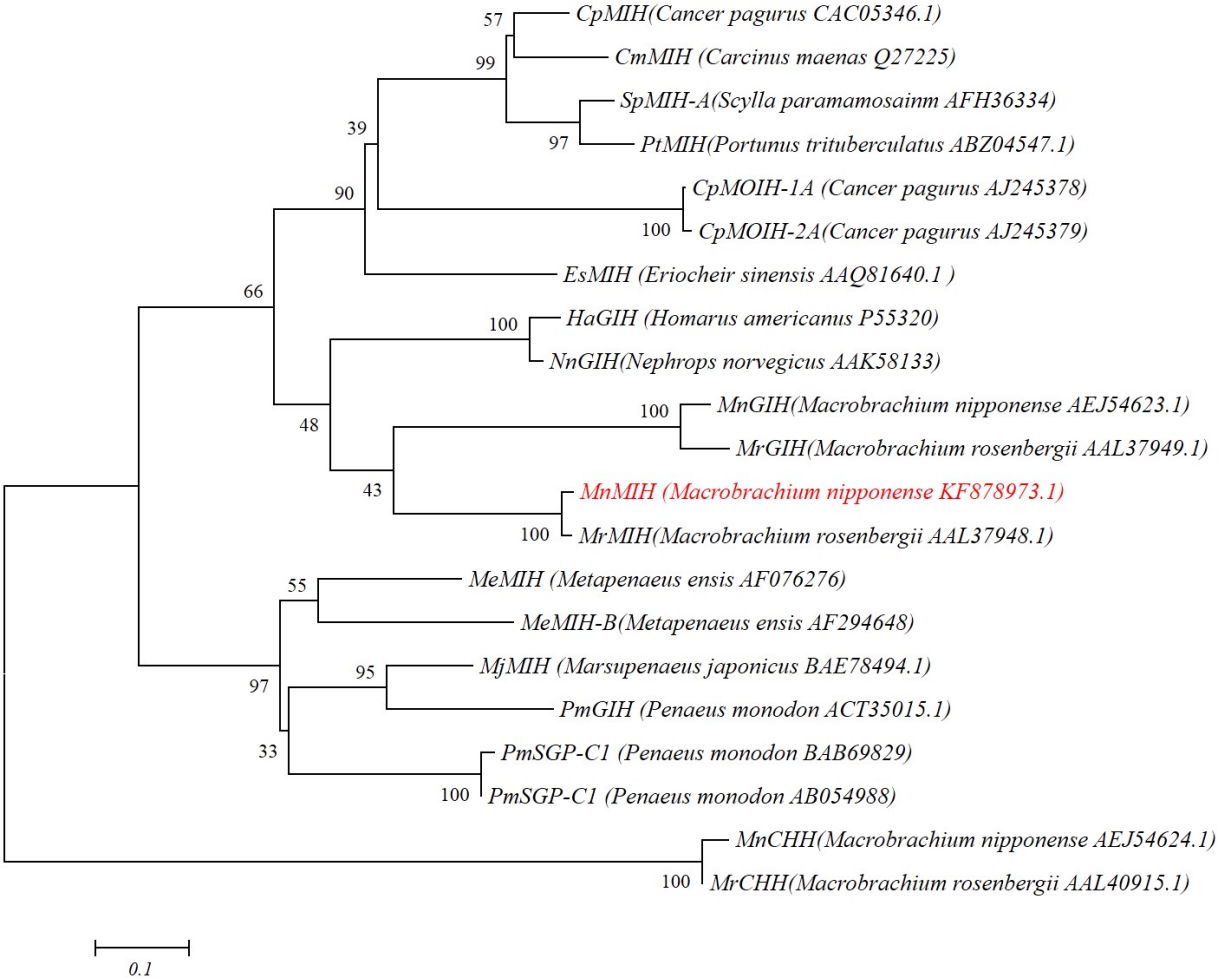
Phylogenetic analysis of mature peptide sequences of CHH family members. Notes: The diagram was generated by the neighbor-joining method using the MEGA 5.0 program. Bootstrapping replications were 1000. GenBank accession numbers are in brackets. *M*. *nipponense* MIH is marked with red color.

To evaluate the evolutionary relationship of MIHs in different species, we conducted a phylogenetic analysis using the NCBI databases, based on the nucleotide sequences of CHHs from 10 species (Fig 2). Mn-MIH showed the highest amino acid identity (95%) with the MIH of *M*. *rosenbergii*, followed by *Portunus pelagicus* (64%) and *Portunus*. *trituberculatus* (60%). The result showed that mature Mn-MIH showed the highest amino acid identity (95%) with the MIH of *M*. *rosenbergii* and they shared almost the same sequence except 6 amino acids. Five different amino acids distribute in signal peptide and only one in mature peptide. *M*. *nipponense* and *M*. *rosenbergii*, which belong to Palaemonidae, have closest relationship. In our previous studies of CHH gene family, mature GIH showed 93% amino acid identity and mature CHH shared 99% amino acid identity in this two spices [8]. To study the relationship of Mn-MIH with subtype II neuropeptide of other shrimp species, type II sequences of other crustacean were selected for analysis, but were restricted to decapods (crab, lobster, shrimp, and crayfish) (Fig 3). A condensed phylogenetic tree was constructed using MEGA 5.0, based on the neighbor-joining method using complete GIHs, MIHs, MOIH (mandibular organ inhibiting hormone), and CHHs proteins deposited in NCBI.Compared to other decapods, Mn-MIH was more closely related to the Mr-MIH of *M*. *rosenbergii*. Mn-MIH and Mr-MIH were more related to the clusters of Mn-GIH and Mr-GIH. Compared to other crustaceans, Mn-MIH shared a higher percentage of amino acid sequence identity with fresh water prawn, lobster, and crab, than with marine shrimps.

### The expression pattern of Mn-MIH

**Fig 4:**
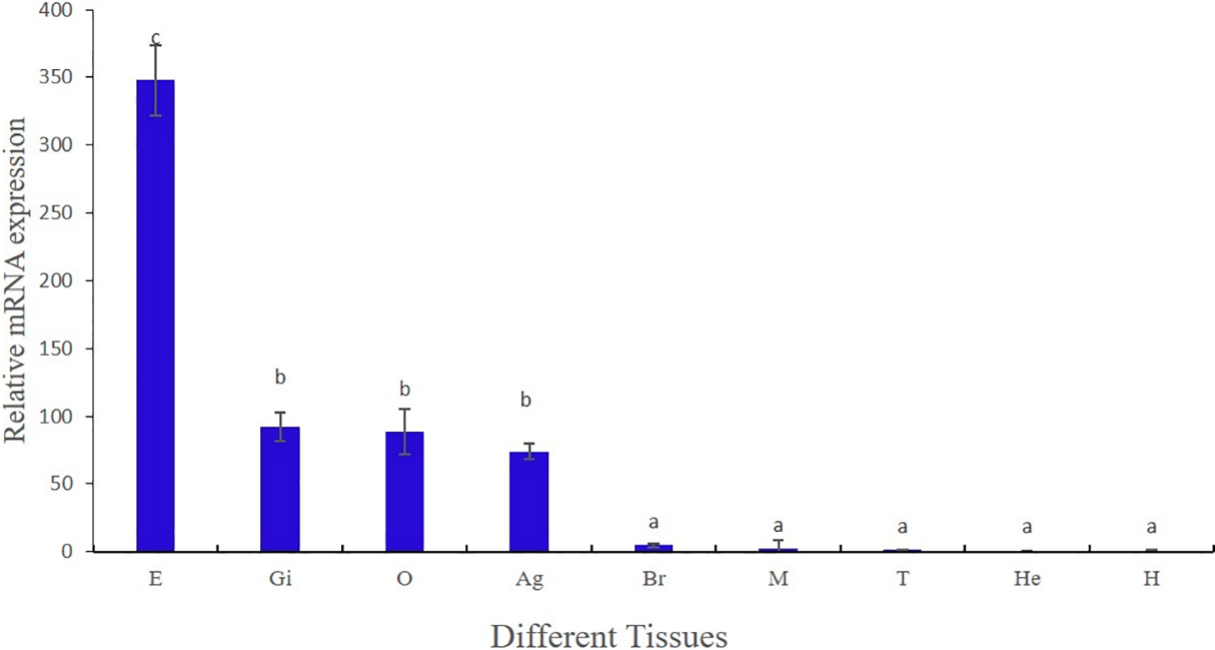
Mn-MIH expression in different tissues of *M*. *Nipponense*. Notes: Tissues were labeled as following: Notes: E: eyestalk; Gi: gill; O: ovary; Ag: abdominal ganglion Br: brain; M: muscle; T: testis; He: hepatopancreas; H: heart. It was normalized to the Mn-β-Actin transcript level. Data were shown as means ± SD (standard deviation) of three separate individuals in the tissues. Bars with different letters were significantly different (P < 0.05).

To evaluate the expression levels of Mn-MIH in different tissues of adult oriental river prawns, mRNA expression levels were quantified in various tissues including hepatopancreas, muscle, ovary, testis, gill, eyestalk, abdominal ganglion, heart, and brain. As shown in Fig 4, the highest mRNA expression was observed in the eyestalk (P < 0.05). Relatively high mRNA expression was also observed in ovary, gills, and abdominal ganglion (P < 0.05). Mn-MIH was weakly expressed in brain, muscle, and testis. Almost no mRNA expression was detected in hepatopancreas and heart.

**Fig 5:**
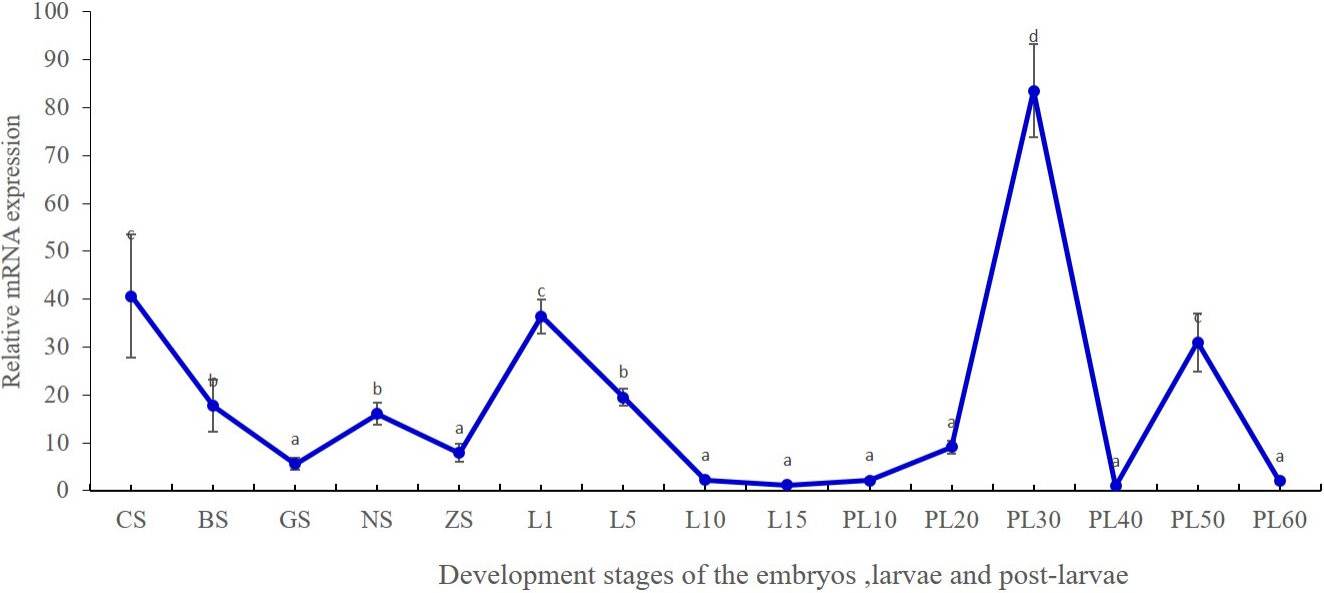
The temporal expression of Mn-MIH transcripts in different stages of embryo, larvae and post-larvae. Notes: The amount of Mn-MIH mRNA was normalized to the β-actin transcript level. Data were shown as means ± SD of three repeated samples during the larvae and post-larvae. CS-cleavage stage; BS-blastula stage; GS-gastrula stage; NS-nauplius stage; ZS-zoea stage. LI- the first day after hatching, PL10- the tenth day post-larvae after larvae, and so on.

The expression of Mn-MIH at different development stages of oriental river prawns was also determined. The developmental stage of embryo was classified into five stages, according to the criteria of Chen [19]. Mn-MIH was expressed in all *M*. *nipponense* embryo developmental stages (Fig 5). During early embryonic development, Mn-MIH had a relative high expression at the cleavage stage (CS), but had a sharp decrease from the CS to the gastrula stage (P < 0.05). Mn-MIH was expressed at a low level until nauplius stage. During larval development, Mn-MIH expression increased significantly after hatching (L1), and then declined gradually to the lowest level, from L5 to L15 in the larval stage (P > 0.05). After metamorphosis, the larvae went into post-larvae stages. Mn-MIH expression exhibited a periodic fluctuation approximately every 20 days (P < 0.05).

**Fig 6:**
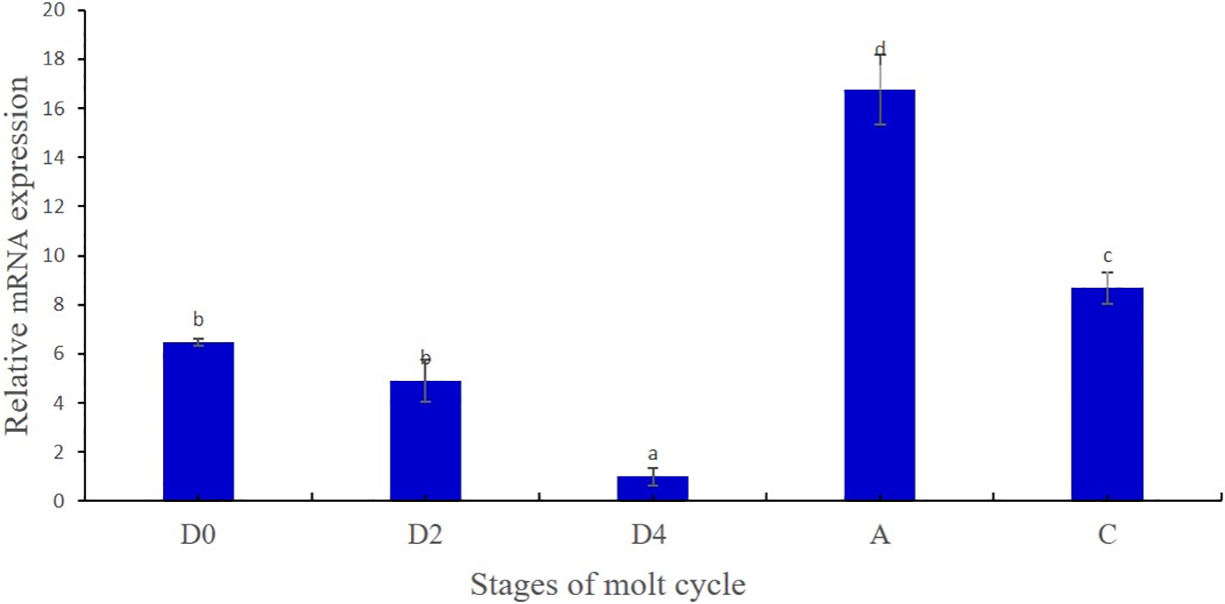
Mn-MIH expression during the molting cycle. Notes: D0-early pre-molt; D2-middle pre-molt; D4-late pre-molt; A-post-molt; C-inter-molt. Data were shown as means ± SD (standard deviation) of three separate individuals in the tissues. Bars with different letters were significantly different (P < 0.05).

We analyzed the transcription profile of Mn-MIH during molting cycles. The different molt stages of the prawn were identified based on the criteria described by Nakatsuji [20]. Each molt cycle of crustaceans is composed of different stages: D_0_ (early pre-molt), D_2_ (middle pre-molt), D_4_ (late pre-molt), A (post-molt), and C (intermolt). Expression of Mn-MIH in the eyestalk in different molting cycles is shown in Fig 6. During each molting cycle, Mn-MIH expression decreased gradually during the pre-molt stage (from early pre-molt to late pre-molt) and reached the lowest levels at the late pre-molt stage (D_4_, late pre-molt) (P > 0.05). Mn-MIH expression then increased rapidly to the highest level after molting (A, post-molt) (P < 0.01); however there was a lower expression in intermolting stage (C, intermolt).

### Functional analysis of Mn-MIH

**Fig 7:**
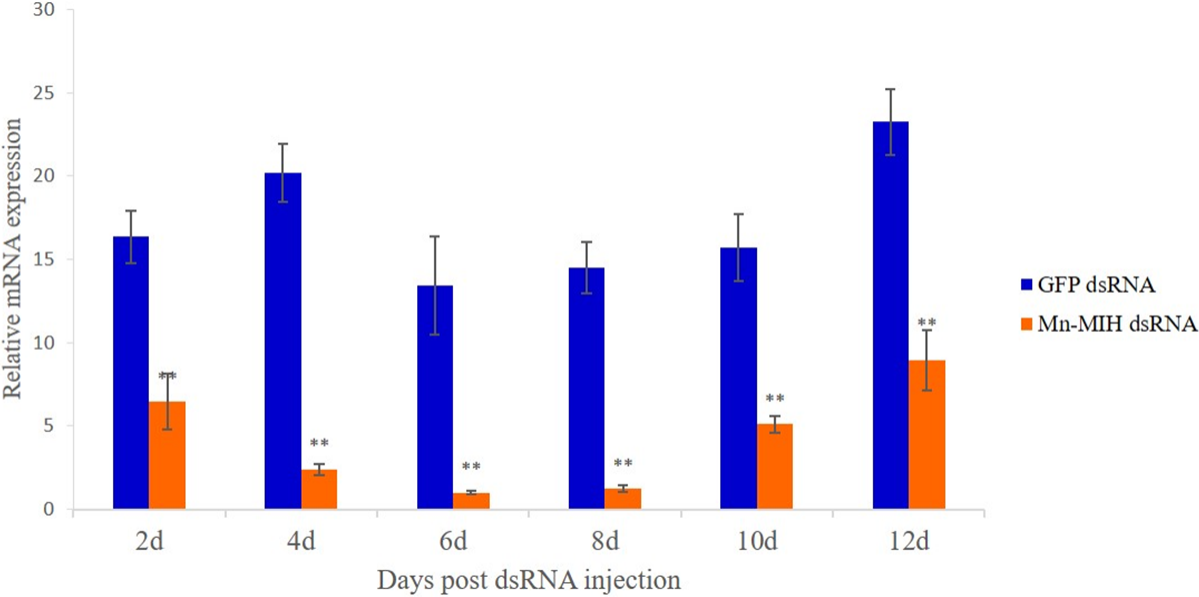
Efficiency of RNAi-mediated knockdown in eyestalks of *M*. *Nipponense*. Different letters denote significant differences (P < 0.05). Error bars represent the mean± standard error.

To investigate the involvement of Mn-MIH in molting and growth, the expression level of Mn-MIH in the eyestalk was determined by qRT-PCR; the changes in molting times and body weight were record after RNAi. The results in Fig 7 show that, the RNAi knockdown of Mn-MIH was successful, reaching a 62% down-regulation compared to control animals at 2^nd^ after injection. Mn-MIH transcript levels were down-regulated, reaching the lowest levels at 6^th^ after RNAi (93% down-regulation), and then increased gradually from 8^th^. Mn-MIH transcript levels only increased to 40% of control levels until 12^th^ (P < 0.05), which indicated that the effect of a single RNAi dose at 4μg·g^−1^ b.w. can last for about two weeks.

**Fig 8:**
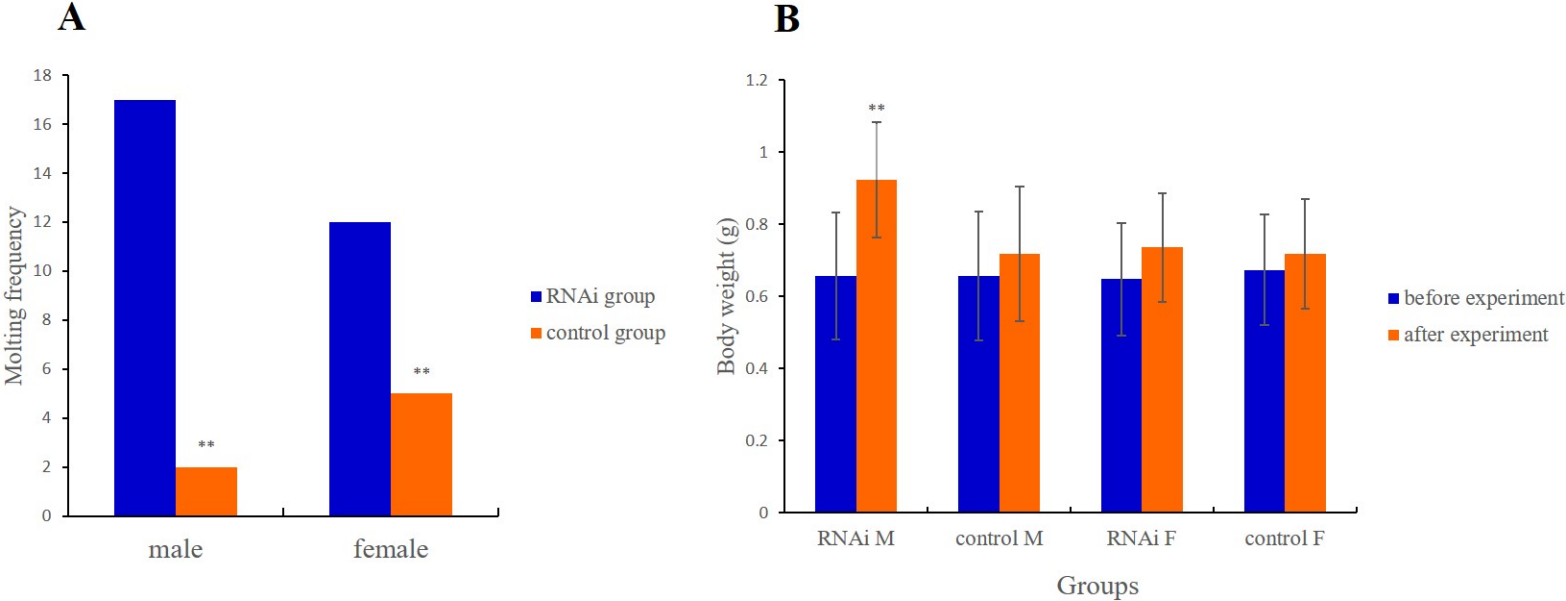
The effects of Mn-MIH knockdown in molting and growth. A: The total number of molting shells and gain weight in different groups during the RNA interference. Notes: ** means significantly different (P < 0.01). B: The average body weight changes in male and female groups during the RNA interference. Notes: M means male, F means female; ** means significantly different (P < 0.01).

Long-term RNAi treatment stably reduced Mn-MIH expression to a low level for 6 weeks after RNAi, and significantly shortened the molting cycles. In males, a total of 17 and 2 shells were found in experimental and control groups, respectively (P < 0.05). In females, the shell numbers were 12 and 5 in experimental and control groups, respectively (P < 0.05) (Fig 8A). Body weight of all the prawns in both experimental and control groups were measured before and after RNAi. After 6 weeks, the body weight of male prawns in the RNAi group increased significantly more than the body weight of the control group (0.26 g and 0.06 g, respectively; P < 0.01). However, the body weight of female prawns showed no differences between both groups (0.08 g and 0.04 g, respectively; P > 0.05) (Fig 8B).

## Discussion

In recent years, various studies reported the cloning and characterization of CHH family members from different crustaceans; however, knowledge on the classification and functions of these neuropeptides is still incomplete. In this study, we obtained a full length cDNA of MIH from the eyestalk of *M*. *nipponense*. No CHH precursor-related peptide was present between the signal peptide and the mature hormone peptide, indicating that the Mn-MIH protein belongs to the CHH type II family. CHH family neuropeptides have high similarity, and the precise classification of a newly discovered sequence is complicated. This study also revealed that MIHs were closely related to GIHs, which suggests that they may have common functions. CHH proteins were separated from GIHs and MIHs, which are more specialized functionally. Phylogenetic tree analysis showed that all the selected crustacean MIHs were divided into two clades: fresh water spices, and marine spices. This separation indicated the evolutionary paths of MIHs. Thus, more and complete researches are in need to clarify the true function of the CHH family genes and establish the evolutionary relationship among the neuropeptides.

In earlier studies MIHs were found exclusively in eyestalk [12, 21–22], but recent reports detected MIHs in other non-neuronal tissues, such as brain, sexual gland, thoracic ganglion, and abdominal ganglion [7, 23–24]. In this paper, we detected MIH not only in neuronal tissues and in the ovary, but also in the gills of *M*. *nipponense*. The presence of Mn-MIH in the ovary might be evidence of its inhibition capacity. The presence of Mn-MIH in other tissues suggests the existence of functions that affect molting and/or other physiological processes. In addition, we found clear differences in the expression of Mn-MIH between sexes. Mn-MIH had a relatively high expression level in the ovary, but almost no expression in the testis. Recent reports indicate that MIH is involved in the regulation of vitellogenesis and in the expression of insulin-like androgenic gland hormone (IAG) in mature female and male crustaceans [7, 17, 25]. Further studies are needed to fully understand the regulatory roles of MIHs in crustacean reproduction.

Earlier reports indicated that MIH was not expressed during embryonic development [26–27]. In this study, we monitored the change of Mn-MIH expression throughout all the developmental stages of *M*. *nipponense*, including embryo, larvae, and post-larvae, and explored Mn-MIH expression patterns during development. Mn-MIH was highly expressed in the CS and then decreased in subsequent embryo stages, suggesting a maternal deposit in the embryos. With the emergence of the eyes, Mn-MIH expression began to increase and reached a peak on the hatching day (L1). After hatching, Mn-MIH expression was gradually down-regulated to the lowest when the larva entered into metamorphosis (L15).

In crustaceans, metamorphosis can be considered as a special ecdysis, which results in an alteration to the whole body-plan during specific stages. MIH is predicted to suppress ecdysone production [28–29]. After metamorphosis, Mn-MIH expression levels changed regularly, in line with the molting cycle, indicating the role of Mn-MIH in growth and development of *M*. *nipponense*. The expression profile of Mn-MIH in the embryo and larva developmental stages indicate its continuous role in maintaining body phase during the lifetime of *M*. *nipponense*.

In vitro and in vivo studies on several species of adult crustaceans have indicated that MIH delays molting [15, 19, 30]. However, the profile of MIH gene expression for molting regulation remains controversial. Previous reports showed that MIH expression in the eyestalk in *Penaeus japonicas* and *Carcinus maenas* did not change during the molting cycle, suggesting that the regulatory role of MIH was post-transcriptional [31–32]. In our study Mn-MIH expression changed significantly with the molting cycle, suggesting that Mn-MIH was involved in negatively regulating ecdysteroidogenesis. These results are similar to a previous study on the green mud crab *Scylla paramamosain* [7]. Our results thus indicate that MIH plays an important role in the regulation of molting. In a few crustaceans, the function of MIH as a molt inhibitor was previously demonstrated through removal of the eyestalk and by MIH injections [3]. MIH RNAi in *Cherax quadricarinatus* led to a significant reduction (up to 32%) in molting intervals, and to an increase in molt mineralization index, which resulted in an acceleration of molt cycles [12]. In Chinese mitten crab *Eriocheir sinensis*, in vitro incubation of Y-organs with a recombinant MIH protein inhibited ecdyseroidogenesis and secretion of ecdysteroids [33]. In this study, MIH RNAi led a significant acceleration of the molt cycles in both males and females of *M*. *nipponense*, which confirmed its regulatory role in crustacean molting.

We investigated the role of Mn-MIH in the regulation of the growth of *M*. *nipponense*. In crustaceans, growth occurs through periodical molting, which is under the control of neuropeptide hormones and ecdysteroids. We recorded the body weight changes of *M*. *nipponense* after Mn-MIH RNAi. Interestingly, there were differences of body weight changes between males and females in the same condition. After Mn-MIH RNAi, males gained weight significantly, while body weight in females remained almost unchanged. We believe that there may be two reasons for this difference. First, the oriental river prawns have great growth differences between males and females. Males grow faster and gain more weight at harvest time than females. Moreover, in *M*. *nipponense* females, growth and reproduction are accompanied by molting, and females cannot spawn until the shell is shed off. During breeding seasons the female prawns enter into a rapid development period with a short reproductive cycle, which affects the growth of female prawns, resulting in individual miniaturization. We hypothesize that in breeding seasons, or under suitable conditions for reproduction, females may spend most energy on reproduction. In *Metapenaeus ensis* and *S*. *paramamosain*, MIH participate in gonad regulation [7, 16, 34]. Further studies are needed to clarify the correlation between MIH and growth and reproduction in *M*. *nipponense*.

## Conclusion

In conclusion, we obtained a molt-inhibiting hormone gene (Mn-MIH) in *M*. *nipponense* and characterized its expression patterns in different tissues, development stages and molting cycles. Besides, the results of RNAi using specific dsRNA validate its acceleration function on molt and growth by body weight monitoring. This study advances our understanding of the biological functions of the Mn-MIH genes and provides new insights into the expression and function of Mn-MIH. It would be very interesting to further elucidate the mechanism of Mn-MIH in the regulation of growth and reproduction.
